# Di-μ-thio­cyanato-bis­{[1,2-bis­(diiso­propyl­phosphan­yl)-1,2-dicarba-*closo*-dodeca­borane]silver(I)}

**DOI:** 10.1107/S1600536810049263

**Published:** 2010-12-04

**Authors:** Liguo Yang, Chengchen Zhu, Dacheng Li

**Affiliations:** a School of Chemistry and Chemical Engineering, Liaocheng University, Shandong 252059, People’s Republic of China

## Abstract

The title compound, [Ag_2_(NCS)_2_(C_14_H_38_B_10_P_2_)_2_], was synthesized by the reaction of 1,2-bis­(diiso­propyl­phos­phan­yl)-1,2-dicarba-*closo*-dodeca­borane with AgSCN. The diisopropyl­phosphanyl-*closo*-carborane ligand is coordinated in a bidentate manner to the Ag^I^ atom through the two P atoms. The coordination of the Ag^I^ atom is distorted tetra­hedral, in which two vertices are formed by the P atoms of the chelating diphosphine ligand, and the other two are occupied by the S and N atoms of the two bridging thio­cyanate anions, leading to a centrosymmetric binuclear complex. The distance between the two C atoms in the carborane skeleton is 1.851 (6) Å.

## Related literature

For related structures, see: Zhang *et al.* (2006 ▶); Paavola *et al.* (2002 ▶, 2002*a* ▶,*b* ▶). For the synthesis and structure of 1,2-bis­(di­isopropyl­phosphan­yl)-1,2-dicarba-*closo*-dodeca­borane, see: Kivekäs *et al.* (1995 ▶).
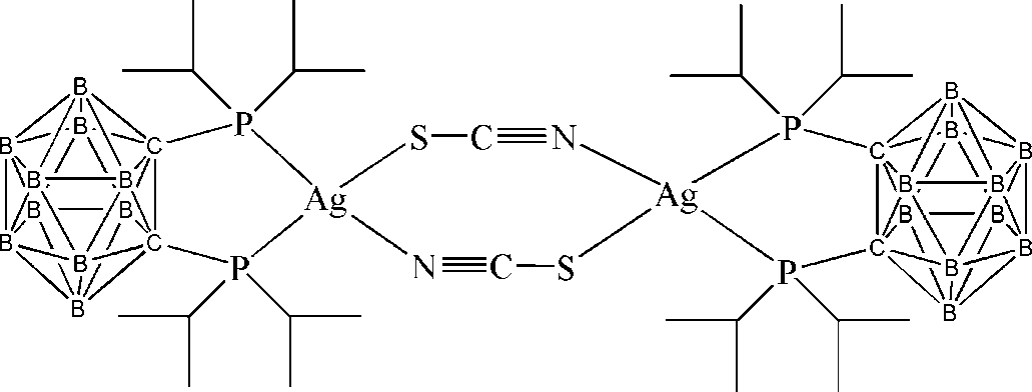


## Experimental

### 

#### Crystal data

[Ag_2_(NCS)_2_(C_14_H_38_B_10_P_2_)_2_]
*M* *_r_* = 1084.87
Monoclinic, 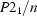

*a* = 7.8075 (9) Å
*b* = 34.220 (3) Å
*c* = 10.6886 (12) Å
β = 110.074 (1)°
*V* = 2682.2 (5) Å^3^
*Z* = 2
Mo *K*α radiation
μ = 0.95 mm^−1^
*T* = 298 K
0.41 × 0.18 × 0.08 mm

#### Data collection

Bruker SMART 1000 CCD diffractometer
Absorption correction: multi-scan (*SADABS*; Sheldrick, 1996 ▶) *T*_min_ = 0.696, *T*_max_ = 0.928
13386 measured reflections
4714 independent reflections
3238 reflections with *I* > 2σ(*I*)
*R*_int_ = 0.050

#### Refinement

*R*[*F* ^2^ > 2σ(*F* ^2^)] = 0.052
*wR*(*F* ^2^) = 0.099
*S* = 1.06
4714 reflections
279 parameters
2 restraints
H-atom parameters constrained
Δρ_max_ = 0.82 e Å^−3^
Δρ_min_ = −0.92 e Å^−3^

### 

Data collection: *SMART* (Siemens, 1996 ▶); cell refinement: *SAINT* (Siemens, 1996 ▶); data reduction: *SAINT*; program(s) used to solve structure: *SHELXS97* (Sheldrick, 2008 ▶); program(s) used to refine structure: *SHELXL97* (Sheldrick, 2008 ▶); molecular graphics: *SHELXTL* (Sheldrick, 2008 ▶); software used to prepare material for publication: *SHELXTL*.

## Supplementary Material

Crystal structure: contains datablocks I, global. DOI: 10.1107/S1600536810049263/gk2319sup1.cif

Structure factors: contains datablocks I. DOI: 10.1107/S1600536810049263/gk2319Isup2.hkl

Additional supplementary materials:  crystallographic information; 3D view; checkCIF report

## Figures and Tables

**Table:** 

Ag1—N1	2.251 (5)
Ag1—P1	2.4566 (14)
Ag1—P2	2.4981 (14)
Ag1—S1	2.5693 (17)

**Table:** 

P1—Ag1—P2	90.97 (4)
N1—Ag1—S1	97.27 (14)

**Fig. 1.**
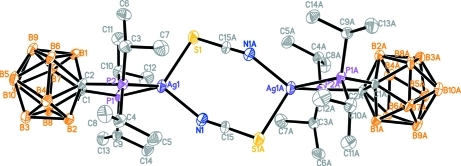
The molecular structure of the title compound with atom labels and 40% probability displacement ellipsoids for non-H atoms. Symmetry code for atoms with the A label: -x + 1,-y,-z + 2. H atoms have been omitted for clarity.

## supplementary crystallographic information

### Comment

The synthesis and structure of 1,2-(P^i^Pr_2_)_2_-1,2-C_2_B_10_H_10_ was reported by Kivekäs *et al.* (1995). Since then, only a few complexes of this ligand with Pt(II) and Pd(II) have been described (Paavola *et al.*, 2002, 2002*a*,b). Here we report the structure of this ligand combined with Ag and thiocyanate ion.

As shown in Fig. 1, the coordination of the Ag atom is distorted tetrahedral, formed by one S atom and one N atom of the two SCN anions and the P atoms of diisopropylphosphanyl-*closo*-carborane ligand (Table 1). The two P—Ag bond lengths are slightly shorter than the corresponding bond lengths in the complex [Ag_2_Cl_2_(C_26_H_30_B_10_P_2_)_2_].2CH_2_Cl_2_ [2.5052 (14) Å; Zhang *et al.*, 2006]). The P—Ag—P angle is slightly larger than the corresponding value of 89.80 Å for the complex [Ag_2_Cl_2_(C_26_H_30_B_10_P_2_)_2_].2CH_2_Cl_2_ (Zhang *et al.*, 2006). The five-memebered chelate ring fomed by the silver atom, two phosphorus atoms and two carbon atoms of the carborane skeleton is strongly flattened with a maximum deviation of 0.322 Å for C2. The torsion angle P1—C1—C2—P2 is -0.4 (5)°, *viz*. smaller than that of 12.1 (2)° in the free ligand (Kivekäs *et al.*, 1995).

### Experimental

The title compound was synthesizd by the reaction of 1 mmol AgSCN and 1 mmol 1,2-(P^i^Pr_2_)_2_-1,2-C_2_B_10_H_10_ in 10 ml dichloromethane under the protection of N_2_, refluxed for 4 h, then a colorless solution formed, and crystals suitable for X-ray diffraction were obtained from a dichloromethane- n-hexane solution.(61.7%, m.p. 553–558 K). FTIR (KBr) *v* (cm^-l^): 2989, 2966, 2930, 2872 (C—H); 2614, 2602, 2585, 2556 (B—H); 1071 (C—P).

### Refinement

All H atoms were placed geometrically and treated as riding on their parent atoms, with B—H 1.10, C—H 0.96 (methyl), C—H 0.98 Å (isopropyl), with *U*_iso_(H) = 1.2*U*_eq_(B), *U*_iso_(H) = 1.5*U*_eq_(C). A rigid bond restraints were applied to the Uij values of Ag1,P1 and Ag1,P2 atoms *via* DELU instruction of SHELXL97 (Sheldrick, 2008).

### Crystal data

**Table:**

[Ag_2_(NCS)_2_(C_14_H_38_B_10_P_2_)_2_]	*F*(000) = 1112
*M**_r_* = 1084.87	*D*_x_ = 1.343 Mg m^−^^3^
Monoclinic, *P*2_1_/*n*	Mo *K*α radiation, λ = 0.71073 Å
Hall symbol: -P 2yn	Cell parameters from 3831 reflections
*a* = 7.8075 (9) Å	θ = 2.4–25.8°
*b* = 34.220 (3) Å	µ = 0.95 mm^−^^1^
*c* = 10.6886 (12) Å	*T* = 298 K
β = 110.074 (1)°	Block, yellow
*V* = 2682.2 (5) Å^3^	0.41 × 0.18 × 0.08 mm
*Z* = 2	

### Data collection

**Table:**

Bruker SMART 1000 CCD diffractometer	4714 independent reflections
Radiation source: fine-focus sealed tube	3238 reflections with *I* > 2σ(*I*)
graphite	*R*_int_ = 0.050
φ and ω scans	θ_max_ = 25.0°, θ_min_ = 2.1°
Absorption correction: multi-scan (*SADABS*; Sheldrick, 1996)	*h* = −8→9
*T*_min_ = 0.696, *T*_max_ = 0.928	*k* = −36→40
13386 measured reflections	*l* = −12→10

### Refinement

**Table:**

Refinement on *F*^2^	Primary atom site location: structure-invariant direct methods
Least-squares matrix: full	Secondary atom site location: difference Fourier map
*R*[*F*^2^ > 2σ(*F*^2^)] = 0.052	Hydrogen site location: inferred from neighbouring sites
*wR*(*F*^2^) = 0.099	H-atom parameters constrained
*S* = 1.06	*w* = 1/[σ^2^(*F*_o_^2^) + (0.0185*P*)^2^ + 6.4963*P*] where *P* = (*F*_o_^2^ + 2*F*_c_^2^)/3
4714 reflections	(Δ/σ)_max_ = 0.001
279 parameters	Δρ_max_ = 0.82 e Å^−^^3^
2 restraints	Δρ_min_ = −0.92 e Å^−^^3^

### Fractional atomic coordinates and isotropic or equivalent isotropic displacement parameters (Å^2^)

**Table:**

	*x*	*y*	*z*	*U*_iso_*/*U*_eq_	
Ag1	0.61123 (6)	0.074944 (11)	0.96399 (4)	0.04357 (15)	
P1	0.77035 (19)	0.13609 (4)	1.05474 (12)	0.0319 (3)	
P2	0.62319 (19)	0.09058 (4)	0.73885 (13)	0.0335 (3)	
S1	0.2929 (2)	0.06561 (4)	0.9800 (2)	0.0638 (5)	
C2	0.7012 (7)	0.14225 (13)	0.7460 (4)	0.0309 (12)	
N1	0.7232 (7)	0.01564 (15)	1.0410 (5)	0.0634 (16)	
C1	0.7792 (7)	0.16616 (13)	0.9114 (5)	0.0311 (12)	
C3	0.4009 (7)	0.08761 (15)	0.6024 (5)	0.0443 (14)	
H3A	0.4158	0.0956	0.5187	0.053*	
C9	1.0151 (7)	0.13069 (15)	1.1608 (5)	0.0398 (13)	
H9A	1.0841	0.1309	1.0995	0.048*	
B1	0.5741 (8)	0.17712 (16)	0.7899 (5)	0.0327 (14)	
H1	0.4439	0.1695	0.8022	0.039*	
B2	0.9279 (8)	0.15162 (17)	0.8327 (6)	0.0342 (14)	
H2	1.0248	0.1276	0.8725	0.041*	
C4	0.7877 (8)	0.06395 (14)	0.6774 (5)	0.0423 (14)	
H4A	0.9030	0.0783	0.7119	0.051*	
B3	0.9813 (9)	0.19488 (18)	0.7612 (6)	0.0420 (16)	
H3	1.1164	0.1997	0.7530	0.050*	
B4	0.8264 (9)	0.16041 (18)	0.6591 (6)	0.0401 (16)	
H4	0.8620	0.1431	0.5848	0.048*	
B5	0.7755 (9)	0.21104 (18)	0.6369 (6)	0.0469 (18)	
H5	0.7765	0.2270	0.5478	0.056*	
C10	0.6616 (8)	0.16743 (16)	1.1476 (5)	0.0459 (15)	
H10A	0.7314	0.1917	1.1744	0.055*	
B6	0.6018 (9)	0.17690 (17)	0.6309 (6)	0.0371 (15)	
H6	0.4882	0.1708	0.5381	0.044*	
B7	0.7247 (9)	0.21373 (16)	0.8895 (6)	0.0379 (16)	
H7	0.6898	0.2313	0.9636	0.045*	
B8	0.9487 (9)	0.19743 (16)	0.9160 (6)	0.0370 (15)	
H8	1.0633	0.2041	1.0077	0.044*	
B9	0.6147 (10)	0.22145 (17)	0.7165 (6)	0.0440 (17)	
H9	0.5083	0.2439	0.6792	0.053*	
C11	0.4625 (8)	0.17704 (17)	1.0711 (6)	0.0542 (16)	
H11A	0.3993	0.1537	1.0310	0.081*	
H11B	0.4556	0.1958	1.0029	0.081*	
H11C	0.4072	0.1877	1.1312	0.081*	
C5	0.8260 (9)	0.02295 (16)	0.7408 (6)	0.0586 (18)	
H5A	0.7171	0.0074	0.7089	0.088*	
H5B	0.8637	0.0253	0.8359	0.088*	
H5C	0.9209	0.0106	0.7169	0.088*	
B10	0.8498 (9)	0.23347 (17)	0.7945 (6)	0.0426 (17)	
H10	0.8978	0.2639	0.8068	0.051*	
C12	0.6691 (9)	0.1440 (2)	1.2724 (5)	0.069 (2)	
H12A	0.6220	0.1596	1.3277	0.104*	
H12B	0.7932	0.1369	1.3213	0.104*	
H12C	0.5968	0.1207	1.2460	0.104*	
C6	0.2489 (8)	0.11169 (17)	0.6243 (6)	0.0529 (16)	
H6A	0.1335	0.1040	0.5607	0.079*	
H6B	0.2694	0.1389	0.6131	0.079*	
H6C	0.2481	0.1073	0.7128	0.079*	
C7	0.3449 (9)	0.04416 (17)	0.5935 (6)	0.0640 (19)	
H7A	0.3260	0.0366	0.6742	0.096*	
H7B	0.4397	0.0284	0.5813	0.096*	
H7C	0.2340	0.0405	0.5193	0.096*	
C8	0.7401 (9)	0.06209 (17)	0.5261 (6)	0.0611 (18)	
H8A	0.8417	0.0517	0.5059	0.092*	
H8B	0.7129	0.0879	0.4894	0.092*	
H8C	0.6357	0.0455	0.4883	0.092*	
C13	1.0984 (8)	0.16309 (17)	1.2634 (5)	0.0591 (18)	
H13A	1.0457	0.1620	1.3324	0.089*	
H13B	1.0736	0.1881	1.2202	0.089*	
H13C	1.2278	0.1593	1.3018	0.089*	
C15	0.7150 (8)	−0.01747 (17)	1.0327 (6)	0.0467 (15)	
C14	1.0478 (8)	0.09062 (17)	1.2272 (6)	0.0565 (17)	
H14A	1.1762	0.0867	1.2715	0.085*	
H14B	1.0012	0.0707	1.1608	0.085*	
H14C	0.9864	0.0892	1.2910	0.085*	

### Atomic displacement parameters (Å^2^)

**Table:**

	*U*^11^	*U*^22^	*U*^33^	*U*^12^	*U*^13^	*U*^23^
Ag1	0.0537 (3)	0.0293 (2)	0.0454 (2)	−0.0062 (2)	0.0139 (2)	0.0046 (2)
P1	0.0414 (9)	0.0266 (7)	0.0250 (7)	−0.0005 (6)	0.0079 (6)	0.0023 (5)
P2	0.0384 (9)	0.0254 (7)	0.0320 (6)	−0.0020 (6)	0.0060 (6)	−0.0047 (6)
S1	0.0635 (12)	0.0317 (9)	0.1096 (14)	0.0047 (7)	0.0469 (11)	0.0074 (8)
C2	0.040 (3)	0.023 (3)	0.025 (3)	0.001 (2)	0.005 (2)	0.000 (2)
N1	0.051 (4)	0.039 (3)	0.093 (4)	0.003 (3)	0.015 (3)	0.023 (3)
C1	0.035 (3)	0.021 (3)	0.032 (3)	−0.002 (2)	0.005 (2)	0.001 (2)
C3	0.044 (4)	0.039 (3)	0.042 (3)	−0.006 (3)	0.005 (3)	−0.009 (3)
C9	0.039 (3)	0.042 (3)	0.032 (3)	−0.001 (3)	0.004 (3)	0.007 (2)
B1	0.037 (4)	0.032 (3)	0.025 (3)	0.005 (3)	0.006 (3)	0.002 (2)
B2	0.038 (4)	0.029 (3)	0.034 (3)	0.000 (3)	0.011 (3)	−0.001 (3)
C4	0.050 (4)	0.030 (3)	0.045 (3)	−0.001 (3)	0.014 (3)	−0.010 (2)
B3	0.049 (4)	0.035 (4)	0.045 (4)	0.000 (3)	0.021 (3)	0.006 (3)
B4	0.047 (4)	0.043 (4)	0.030 (3)	0.002 (3)	0.012 (3)	0.005 (3)
B5	0.057 (5)	0.039 (4)	0.043 (4)	−0.002 (3)	0.015 (3)	0.017 (3)
C10	0.060 (4)	0.039 (3)	0.036 (3)	−0.002 (3)	0.014 (3)	−0.004 (3)
B6	0.046 (4)	0.035 (3)	0.027 (3)	0.005 (3)	0.008 (3)	0.010 (3)
B7	0.054 (4)	0.016 (3)	0.034 (3)	0.007 (3)	0.003 (3)	−0.001 (2)
B8	0.043 (4)	0.025 (3)	0.039 (4)	−0.010 (3)	0.009 (3)	0.001 (3)
B9	0.056 (5)	0.026 (3)	0.044 (4)	0.004 (3)	0.011 (3)	0.009 (3)
C11	0.064 (4)	0.058 (4)	0.047 (4)	0.007 (3)	0.028 (3)	−0.009 (3)
C5	0.064 (5)	0.038 (4)	0.069 (4)	0.010 (3)	0.017 (4)	−0.006 (3)
B10	0.056 (5)	0.022 (3)	0.050 (4)	−0.003 (3)	0.019 (4)	0.004 (3)
C12	0.088 (5)	0.088 (5)	0.034 (4)	0.016 (4)	0.025 (4)	0.006 (3)
C6	0.039 (4)	0.060 (4)	0.053 (4)	−0.006 (3)	0.006 (3)	−0.005 (3)
C7	0.056 (5)	0.058 (4)	0.066 (4)	−0.017 (3)	0.006 (3)	−0.021 (3)
C8	0.081 (5)	0.053 (4)	0.056 (4)	−0.006 (3)	0.032 (4)	−0.015 (3)
C13	0.057 (4)	0.058 (4)	0.045 (4)	−0.017 (3)	−0.005 (3)	0.001 (3)
C15	0.039 (4)	0.046 (4)	0.058 (4)	0.006 (3)	0.020 (3)	0.026 (3)
C14	0.059 (4)	0.055 (4)	0.044 (4)	0.006 (3)	0.004 (3)	0.017 (3)

### Geometric parameters (Å, °)

**Table:**

Ag1—N1	2.251 (5)	B4—H4	1.1000
Ag1—P1	2.4566 (14)	B5—B10	1.759 (9)
Ag1—P2	2.4981 (14)	B5—B6	1.775 (9)
Ag1—S1	2.5693 (17)	B5—B9	1.778 (10)
P1—C10	1.853 (6)	B5—H5	1.1000
P1—C1	1.866 (5)	C10—C11	1.524 (8)
P1—C9	1.867 (5)	C10—C12	1.541 (7)
P2—C3	1.847 (5)	C10—H10A	0.9800
P2—C2	1.863 (5)	B6—B9	1.763 (8)
P2—C4	1.868 (5)	B6—H6	1.1000
S1—C15^i^	1.652 (6)	B7—B8	1.763 (9)
C2—B4	1.682 (8)	B7—B10	1.767 (9)
C2—B6	1.693 (7)	B7—B9	1.771 (8)
C2—B1	1.717 (7)	B7—H7	1.1000
C2—B2	1.723 (8)	B8—B10	1.765 (8)
C2—C1	1.851 (6)	B8—H8	1.1000
N1—C15	1.137 (7)	B9—B10	1.784 (9)
C1—B7	1.678 (7)	B9—H9	1.1000
C1—B8	1.689 (7)	C11—H11A	0.9600
C1—B1	1.722 (7)	C11—H11B	0.9600
C1—B2	1.725 (8)	C11—H11C	0.9600
C3—C6	1.527 (7)	C5—H5A	0.9600
C3—C7	1.543 (7)	C5—H5B	0.9600
C3—H3A	0.9800	C5—H5C	0.9600
C9—C14	1.525 (7)	B10—H10	1.1000
C9—C13	1.538 (7)	C12—H12A	0.9600
C9—H9A	0.9800	C12—H12B	0.9600
B1—B6	1.786 (8)	C12—H12C	0.9600
B1—B9	1.786 (8)	C6—H6A	0.9600
B1—B7	1.796 (8)	C6—H6B	0.9600
B1—H1	1.1000	C6—H6C	0.9600
B2—B4	1.775 (8)	C7—H7A	0.9600
B2—B3	1.780 (8)	C7—H7B	0.9600
B2—B8	1.783 (8)	C7—H7C	0.9600
B2—H2	1.1000	C8—H8A	0.9600
C4—C8	1.531 (7)	C8—H8B	0.9600
C4—C5	1.542 (7)	C8—H8C	0.9600
C4—H4A	0.9800	C13—H13A	0.9600
B3—B8	1.761 (9)	C13—H13B	0.9600
B3—B4	1.772 (9)	C13—H13C	0.9600
B3—B10	1.782 (9)	C15—S1^i^	1.652 (6)
B3—B5	1.785 (9)	C14—H14A	0.9600
B3—H3	1.1000	C14—H14B	0.9600
B4—B6	1.767 (9)	C14—H14C	0.9600
B4—B5	1.775 (9)		
			
N1—Ag1—P1	122.90 (14)	B9—B5—B3	108.0 (4)
N1—Ag1—P2	114.03 (15)	B10—B5—H5	121.0
P1—Ag1—P2	90.97 (4)	B4—B5—H5	122.2
N1—Ag1—S1	97.27 (14)	B6—B5—H5	121.9
P1—Ag1—S1	116.70 (5)	B9—B5—H5	122.0
P2—Ag1—S1	116.60 (6)	B3—B5—H5	121.8
C10—P1—C1	105.9 (2)	C11—C10—C12	107.8 (5)
C10—P1—C9	107.1 (3)	C11—C10—P1	114.1 (4)
C1—P1—C9	103.6 (2)	C12—C10—P1	105.9 (4)
C10—P1—Ag1	116.2 (2)	C11—C10—H10A	109.6
C1—P1—Ag1	107.57 (15)	C12—C10—H10A	109.6
C9—P1—Ag1	115.41 (17)	P1—C10—H10A	109.6
C3—P2—C2	106.9 (2)	C2—B6—B9	107.3 (4)
C3—P2—C4	105.6 (2)	C2—B6—B4	58.1 (3)
C2—P2—C4	102.9 (2)	B9—B6—B4	108.1 (5)
C3—P2—Ag1	114.4 (2)	C2—B6—B5	106.3 (4)
C2—P2—Ag1	106.46 (15)	B9—B6—B5	60.4 (4)
C4—P2—Ag1	119.45 (17)	B4—B6—B5	60.2 (4)
C15^i^—S1—Ag1	97.4 (2)	C2—B6—B1	59.1 (3)
B4—C2—B6	63.1 (3)	B9—B6—B1	60.4 (3)
B4—C2—B1	113.2 (4)	B4—B6—B1	106.0 (4)
B6—C2—B1	63.1 (3)	B5—B6—B1	107.7 (4)
B4—C2—B2	62.9 (3)	C2—B6—H6	123.3
B6—C2—B2	113.0 (4)	B9—B6—H6	121.1
B1—C2—B2	107.9 (4)	B4—B6—H6	122.6
B4—C2—C1	107.6 (4)	B5—B6—H6	121.8
B6—C2—C1	107.6 (3)	B1—B6—H6	122.4
B1—C2—C1	57.6 (3)	C1—B7—B8	58.7 (3)
B2—C2—C1	57.6 (3)	C1—B7—B10	106.7 (4)
B4—C2—P2	124.9 (4)	B8—B7—B10	60.0 (4)
B6—C2—P2	124.9 (3)	C1—B7—B9	107.4 (4)
B1—C2—P2	117.3 (4)	B8—B7—B9	108.3 (5)
B2—C2—P2	117.4 (3)	B10—B7—B9	60.5 (4)
C1—C2—P2	117.0 (3)	C1—B7—B1	59.3 (3)
C15—N1—Ag1	150.0 (5)	B8—B7—B1	106.6 (4)
B7—C1—B8	63.2 (3)	B10—B7—B1	107.7 (4)
B7—C1—B1	63.8 (3)	B9—B7—B1	60.1 (3)
B8—C1—B1	113.6 (4)	C1—B7—H7	122.9
B7—C1—B2	113.1 (4)	B8—B7—H7	122.3
B8—C1—B2	62.9 (3)	B10—B7—H7	121.8
B1—C1—B2	107.6 (4)	B9—B7—H7	121.2
B7—C1—C2	108.1 (3)	B1—B7—H7	122.3
B8—C1—C2	107.8 (4)	C1—B8—B7	58.1 (3)
B1—C1—C2	57.3 (3)	C1—B8—B3	107.8 (4)
B2—C1—C2	57.5 (3)	B7—B8—B3	108.7 (4)
B7—C1—P1	124.8 (4)	C1—B8—B10	106.3 (4)
B8—C1—P1	125.1 (3)	B7—B8—B10	60.1 (4)
B1—C1—P1	116.9 (3)	B3—B8—B10	60.7 (3)
B2—C1—P1	117.5 (3)	C1—B8—B2	59.5 (3)
C2—C1—P1	116.6 (3)	B7—B8—B2	106.4 (4)
C6—C3—C7	108.1 (5)	B3—B8—B2	60.3 (3)
C6—C3—P2	114.7 (4)	B10—B8—B2	107.7 (4)
C7—C3—P2	105.8 (4)	C1—B8—H8	123.1
C6—C3—H3A	109.4	B7—B8—H8	122.4
C7—C3—H3A	109.4	B3—B8—H8	120.8
P2—C3—H3A	109.4	B10—B8—H8	121.9
C14—C9—C13	110.7 (4)	B2—B8—H8	122.2
C14—C9—P1	110.1 (4)	B6—B9—B7	109.5 (4)
C13—C9—P1	117.1 (4)	B6—B9—B5	60.1 (4)
C14—C9—H9A	106.0	B7—B9—B5	107.5 (5)
C13—C9—H9A	106.0	B6—B9—B10	108.0 (5)
P1—C9—H9A	106.0	B7—B9—B10	59.6 (4)
C2—B1—C1	65.1 (3)	B5—B9—B10	59.2 (4)
C2—B1—B6	57.8 (3)	B6—B9—B1	60.4 (3)
C1—B1—B6	109.4 (4)	B7—B9—B1	60.7 (3)
C2—B1—B9	105.3 (4)	B5—B9—B1	107.5 (4)
C1—B1—B9	104.9 (4)	B10—B9—B1	107.5 (4)
B6—B1—B9	59.2 (3)	B6—B9—H9	120.9
C2—B1—B7	108.9 (4)	B7—B9—H9	121.2
C1—B1—B7	56.9 (3)	B5—B9—H9	122.4
B6—B1—B7	107.4 (4)	B10—B9—H9	122.4
B9—B1—B7	59.3 (3)	B1—B9—H9	121.7
C2—B1—H1	120.8	C10—C11—H11A	109.5
C1—B1—H1	121.1	C10—C11—H11B	109.5
B6—B1—H1	122.1	H11A—C11—H11B	109.5
B9—B1—H1	124.3	C10—C11—H11C	109.5
B7—B1—H1	122.6	H11A—C11—H11C	109.5
C2—B2—C1	65.0 (3)	H11B—C11—H11C	109.5
C2—B2—B4	57.4 (3)	C4—C5—H5A	109.5
C1—B2—B4	109.1 (4)	C4—C5—H5B	109.5
C2—B2—B3	105.8 (4)	H5A—C5—H5B	109.5
C1—B2—B3	105.4 (4)	C4—C5—H5C	109.5
B4—B2—B3	59.8 (3)	H5A—C5—H5C	109.5
C2—B2—B8	109.4 (4)	H5B—C5—H5C	109.5
C1—B2—B8	57.6 (3)	B8—B10—B5	108.5 (4)
B4—B2—B8	107.7 (4)	B8—B10—B7	59.9 (3)
B3—B2—B8	59.2 (3)	B5—B10—B7	108.6 (4)
C2—B2—H2	120.8	B8—B10—B3	59.5 (3)
C1—B2—H2	121.1	B5—B10—B3	60.5 (4)
B4—B2—H2	122.1	B7—B10—B3	107.5 (4)
B3—B2—H2	123.9	B8—B10—B9	107.6 (4)
B8—B2—H2	122.2	B5—B10—B9	60.3 (4)
C8—C4—C5	111.5 (4)	B7—B10—B9	59.8 (3)
C8—C4—P2	116.4 (4)	B3—B10—B9	107.9 (4)
C5—C4—P2	110.0 (4)	B8—B10—H10	121.9
C8—C4—H4A	106.1	B5—B10—H10	121.0
C5—C4—H4A	106.1	B7—B10—H10	121.8
P2—C4—H4A	106.1	B3—B10—H10	122.0
B8—B3—B4	108.9 (4)	B9—B10—H10	121.9
B8—B3—B2	60.5 (3)	C10—C12—H12A	109.5
B4—B3—B2	60.0 (3)	C10—C12—H12B	109.5
B8—B3—B10	59.8 (3)	H12A—C12—H12B	109.5
B4—B3—B10	107.3 (5)	C10—C12—H12C	109.5
B2—B3—B10	107.1 (4)	H12A—C12—H12C	109.5
B8—B3—B5	107.5 (5)	H12B—C12—H12C	109.5
B4—B3—B5	59.9 (4)	C3—C6—H6A	109.5
B2—B3—B5	107.1 (5)	C3—C6—H6B	109.5
B10—B3—B5	59.1 (4)	H6A—C6—H6B	109.5
B8—B3—H3	121.2	C3—C6—H6C	109.5
B4—B3—H3	121.4	H6A—C6—H6C	109.5
B2—B3—H3	122.1	H6B—C6—H6C	109.5
B10—B3—H3	122.6	C3—C7—H7A	109.5
B5—B3—H3	122.5	C3—C7—H7B	109.5
C2—B4—B6	58.8 (3)	H7A—C7—H7B	109.5
C2—B4—B3	108.0 (4)	C3—C7—H7C	109.5
B6—B4—B3	108.7 (5)	H7A—C7—H7C	109.5
C2—B4—B5	106.8 (4)	H7B—C7—H7C	109.5
B6—B4—B5	60.1 (4)	C4—C8—H8A	109.5
B3—B4—B5	60.4 (4)	C4—C8—H8B	109.5
C2—B4—B2	59.7 (3)	H8A—C8—H8B	109.5
B6—B4—B2	107.1 (4)	C4—C8—H8C	109.5
B3—B4—B2	60.2 (3)	H8A—C8—H8C	109.5
B5—B4—B2	107.7 (4)	H8B—C8—H8C	109.5
C2—B4—H4	122.6	C9—C13—H13A	109.5
B6—B4—H4	122.0	C9—C13—H13B	109.5
B3—B4—H4	121.0	H13A—C13—H13B	109.5
B5—B4—H4	121.9	C9—C13—H13C	109.5
B2—B4—H4	122.1	H13A—C13—H13C	109.5
B10—B5—B4	108.2 (4)	H13B—C13—H13C	109.5
B10—B5—B6	108.6 (5)	N1—C15—S1^i^	178.8 (6)
B4—B5—B6	59.7 (3)	C9—C14—H14A	109.5
B10—B5—B9	60.6 (4)	C9—C14—H14B	109.5
B4—B5—B9	107.1 (4)	H14A—C14—H14B	109.5
B6—B5—B9	59.5 (4)	C9—C14—H14C	109.5
B10—B5—B3	60.4 (4)	H14A—C14—H14C	109.5
B4—B5—B3	59.7 (3)	H14B—C14—H14C	109.5
B6—B5—B3	107.7 (4)		
			
N1—Ag1—P1—C10	−111.6 (3)	Ag1—P2—C2—C1	8.3 (3)
P2—Ag1—P1—C10	128.6 (2)	P1—Ag1—N1—C15	−172.8 (9)
S1—Ag1—P1—C10	8.0 (2)	P2—Ag1—N1—C15	−64.7 (10)
N1—Ag1—P1—C1	130.0 (2)	S1—Ag1—N1—C15	58.7 (10)
P2—Ag1—P1—C1	10.24 (17)	P2—C2—C1—P1	−0.4 (5)
S1—Ag1—P1—C1	−110.41 (18)	C10—P1—C1—C2	−132.8 (3)
N1—Ag1—P1—C9	15.0 (3)	C9—P1—C1—C2	114.7 (3)
P2—Ag1—P1—C9	−104.8 (2)	Ag1—P1—C1—C2	−7.9 (4)
S1—Ag1—P1—C9	134.6 (2)	C2—P2—C3—C6	−64.1 (5)
N1—Ag1—P2—C3	104.8 (2)	C4—P2—C3—C6	−173.2 (4)
P1—Ag1—P2—C3	−128.15 (19)	Ag1—P2—C3—C6	53.4 (5)
S1—Ag1—P2—C3	−7.42 (19)	C2—P2—C3—C7	176.9 (4)
N1—Ag1—P2—C2	−137.4 (2)	C4—P2—C3—C7	67.8 (4)
P1—Ag1—P2—C2	−10.35 (17)	Ag1—P2—C3—C7	−65.6 (4)
S1—Ag1—P2—C2	110.38 (17)	C10—P1—C9—C14	103.2 (4)
N1—Ag1—P2—C4	−21.7 (3)	C1—P1—C9—C14	−145.2 (4)
P1—Ag1—P2—C4	105.3 (2)	Ag1—P1—C9—C14	−28.0 (4)
S1—Ag1—P2—C4	−133.9 (2)	C10—P1—C9—C13	−24.5 (5)
C3—P2—C2—B4	−88.9 (4)	C1—P1—C9—C13	87.1 (4)
C4—P2—C2—B4	22.0 (5)	Ag1—P1—C9—C13	−155.7 (4)
Ag1—P2—C2—B4	148.4 (4)	C3—P2—C4—C8	25.7 (5)
C3—P2—C2—B6	−9.6 (5)	C2—P2—C4—C8	−86.2 (4)
C4—P2—C2—B6	101.4 (4)	Ag1—P2—C4—C8	156.3 (3)
Ag1—P2—C2—B6	−132.2 (4)	C3—P2—C4—C5	−102.3 (4)
C3—P2—C2—B1	65.4 (4)	C2—P2—C4—C5	145.8 (4)
C4—P2—C2—B1	176.3 (3)	Ag1—P2—C4—C5	28.3 (4)
Ag1—P2—C2—B1	−57.3 (3)	C1—P1—C10—C11	66.3 (5)
C3—P2—C2—B2	−163.5 (4)	C9—P1—C10—C11	176.3 (4)
C4—P2—C2—B2	−52.6 (4)	Ag1—P1—C10—C11	−53.0 (4)
Ag1—P2—C2—B2	73.8 (4)	C1—P1—C10—C12	−175.3 (4)
C3—P2—C2—C1	130.9 (3)	C9—P1—C10—C12	−65.3 (4)
C4—P2—C2—C1	−118.1 (3)	Ag1—P1—C10—C12	65.4 (4)

Symmetry codes: (i) −*x*+1, −*y*, −*z*+2.
